# Genome-wide screens connect HD82 loss-of-function to purine analog resistance in African trypanosomes

**DOI:** 10.1128/msphere.00363-23

**Published:** 2023-12-21

**Authors:** Anna Trenaman, Michele Tinti, Abdelmadjid Atrih, David Horn

**Affiliations:** 1 The Wellcome Centre for Anti-Infectives Research, School of Life Sciences, University of Dundee, Dundee, United Kingdom; 2 Fingerprints Proteomics Facility, School of Life Sciences, University of Dundee, Dundee, United Kingdom; University at Buffalo, Buffalo, New York, USA

**Keywords:** kinetoplastid, nagana, sleeping sickness, trypanosomatid

## Abstract

Nucleoside analogs have been used extensively as anti-infective agents, particularly against viral infections, and have long been considered promising anti-parasitic agents. These pro-drugs are metabolized by host-cell, viral, or parasite enzymes prior to incorporation into DNA, thereby inhibiting DNA replication. Here, we report genes that sensitize African trypanosomes to nucleoside analogs, including the guanosine analog, ganciclovir. We applied ganciclovir selective pressure to a trypanosome genome-wide knockdown library, which yielded nucleoside mono- and diphosphate kinases as hits, validating the approach. The two most dominant hits to emerge, however, were Tb927.6.2800 and Tb927.6.2900, which both encode nuclear proteins; the latter of which is HD82, a SAMHD1-related protein and a putative dNTP triphosphohydrolase. We independently confirmed that HD82, which is conserved among the trypanosomatids, can sensitize *Trypanosoma brucei* to ganciclovir. Since ganciclovir activity depends upon phosphorylation by ectopically expressed viral thymidine kinase, we also tested the adenosine analog, ara-A, that may be fully phosphorylated by native *T. brucei* kinase(s). Both Tb927.6.2800 and HD82 knockdowns were resistant to this analog. Tb927.6.2800 knockdown increased sensitivity to hydroxyurea, while dNTP analysis indicated that HD82 is indeed a triphosphohydrolase with dATP as the preferred substrate. Our results provide insights into nucleoside/nucleotide metabolism and nucleoside analog metabolism and resistance in trypanosomatids. We suggest that the product of 6.2800 sensitizes cells to purine analogs through DNA repair, while HD82 does so by reducing the native purine pool.

**IMPORTANCE:**

There is substantial interest in developing nucleoside analogs as anti-parasitic agents. We used genome-scale genetic screening and discovered two proteins linked to purine analog resistance in African trypanosomes. Our screens also identified two nucleoside kinases required for pro-drug activation, further validating the approach. The top novel hit, HD82, is related to SAMHD1, a mammalian nuclear viral restriction factor. We validated HD82 and localized the protein to the trypanosome nucleus. HD82 appears to sensitize trypanosomes to nucleoside analogs by reducing native pools of nucleotides, providing insights into both nucleoside/nucleotide metabolism and nucleoside analog resistance in trypanosomatids.

## INTRODUCTION

The African trypanosome, *Trypanosoma brucei*, is a protozoan parasite that causes devastating diseases in both humans and animals, while related trypanosomatids cause other devastating and neglected tropical diseases (1). There has been substantial recent interest in developing nucleoside analogs as anti-trypanosomal therapeutics (2–8), specifically because trypanosomatids, unlike their mammalian hosts, lack the capacity for *de novo* purine biosynthesis (5). Consequently, trypanosomatids are purine auxotrophs that, being incapable of *de novo* purine synthesis, salvage purines from their hosts, for the biosynthesis of nucleic acids.

Nucleoside analogs have been used extensively as anti-infective agents, particularly against viral infections (9), with examples including aciclovir, for the treatment of herpes simplex virus (HSV) infections, ganciclovir (GCV) for the treatment of cytomegalovirus infections, and the “ProTide” drug remdesivir, for the treatment of COVID infections. For the reasons above and because trypanosomatids have efficient mechanisms for nucleoside uptake (10), such analogs are considered promising anti-trypanosomal agents. Indeed, the purine analog, allopurinol, continues to be widely used to treat canine leishmaniasis (11). Pathogenesis in human leishmaniasis can also be enhanced by a leishmania virus such that purine analogs that target the virus could yield therapeutic benefit (12).

Another application of purine analogs in *T. brucei* is for negative selection, in combination with expression of herpes simplex virus thymidine kinase (HSV-TK), which effectively uses both pyrimidines and purines as substrates. Such negative selection has been used to investigate mutagenesis (13), glycosome biogenesis (14), and antigenic variation (15) and for Cre-Lox-based conditional null strategies (16). Ganciclovir is typically used as the HSV-TK substrate and pro-drug (17), and HSV-TK substantially increases sensitivity to ganciclovir when expressed in *T. brucei* (13). *T. brucei* thymidine kinase cannot be used for negative selection as it is essential for viability and is involved in both salvage and *de novo* synthesis (18, 19); it also fails to sensitize cells to ganciclovir, possibly due to low-level expression (20).

Here, we use genome-wide RNA interference (RNAi) library screens to identify *T. brucei* genes that sensitize cells to nucleoside analogs. A primary hit is HD82, a nuclear HD domain protein related to SAMHD1, a human viral restriction factor and triphosphohydrolase. Indeed, *T. brucei* HD82 also appears to be a triphosphohydrolase with dATP as the preferred substrate.

## RESULTS

### A genome-wide *T. brucei* screen for ganciclovir-sensitizing genes

**Fig 1:**
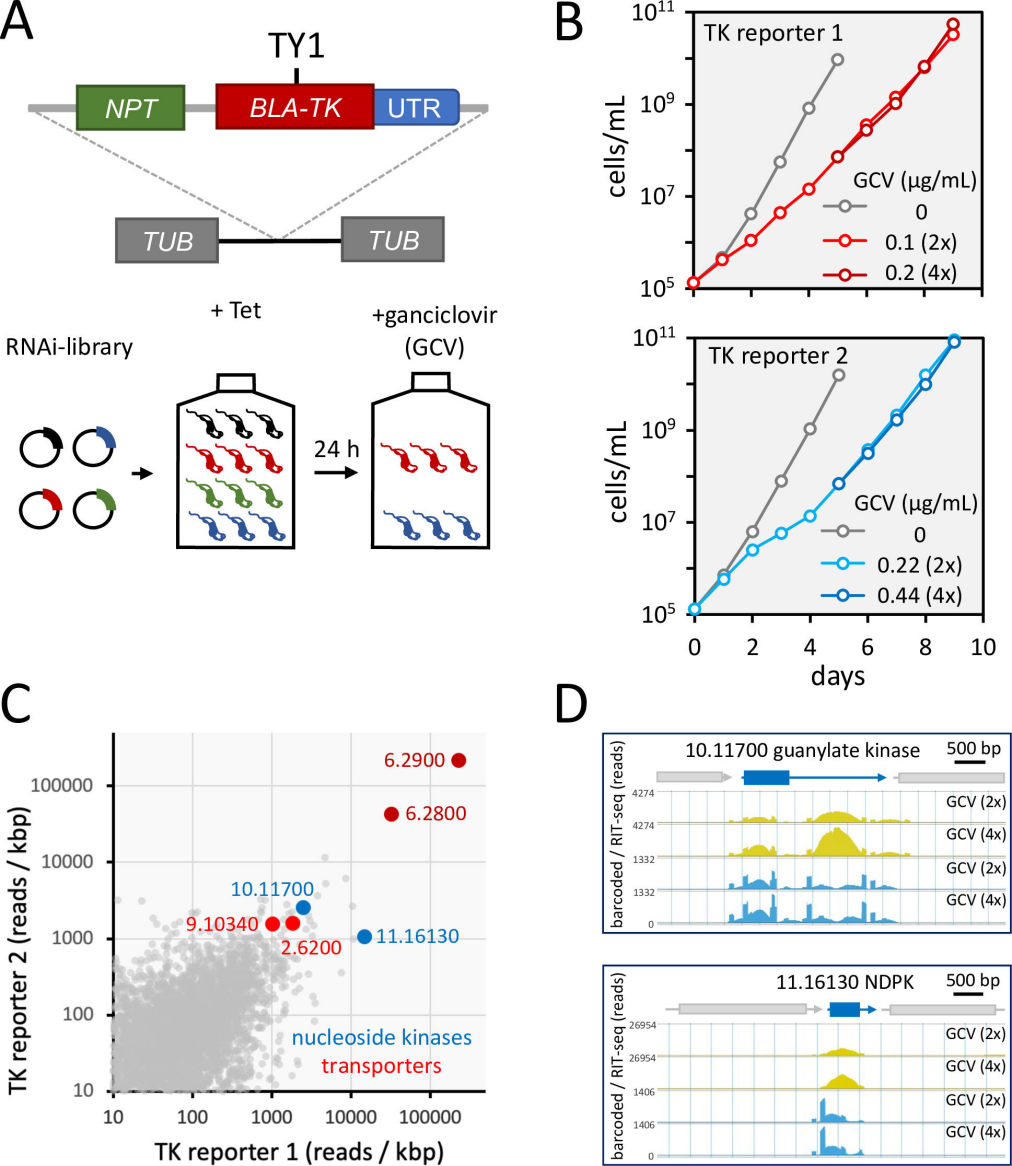
A genome-wide RIT-seq screen using an HSV-TK negative selectable marker. (**A**) Schematic of the reporter construct that was integrated within a *T. brucei* tubulin gene array. The reporter comprises a blasticidin deaminase gene (*BLA*) fused to a herpes simplex virus thymidine kinase gene with a centrally located TY1 epitope tag. The neomycin phosphotransferase gene facilitated recombinant clone selection without applying blasticidin selective pressure. A genome-wide RNAi plasmid library was transfected into the reporter strains, and RIT-seq screens were carried out using GCV selection. (**B**) The cumulative *T. brucei* library population was monitored over 9 days using reporters with two distinct 3′ untranslated regions: (i) *aldolase* and (ii) *VSG*. Tetracycline was added at day 0 to induce knockdown. GCV selection was added at day 1 and the respective effective concentration (EC_50_) was increased from 2× to 4× to generate additional parallel cultures on day 5. Genomic DNA was harvested on day 9. (**C**) RIT-seq read mapping data are compared for all *T. brucei* genes and for both reporters; data from selection with 2× GCV EC_50_ are shown. Notable hits are highlighted. (**D**) RIT-seq read mapping is shown for two nucleoside kinase hits and flanking genes. Total reads (yellow) and reads encompassing the barcode sequence from the RNAi library cassette (blue) are shown; these data are from the screen with reporter 1.

*Trypanosoma brucei* RNAi target sequencing (RIT-seq) screens have been used to identify several drug resistance mechanisms or to identify regulatory factors, using positive selectable marker genes as reporters (1). No such screen has been reported using negative selection, however, with cells expressing a protein that sensitizes cells to a drug. We established a RIT-seq screening assay using a negative selectable marker to identify mechanisms of resistance to the nucleoside analog, GCV. *T. brucei* are sensitized to GCV by expression of the HSV-TK. HSV-TK phosphorylates this thymidine analog (21) which, in its tri-phosphorylated state, acts as a competitive inhibitor of deoxyguanosine triphosphate (dGTP), thereby inhibiting DNA replication. Our screens had the potential to identify other genes that sensitize *T. brucei* to GCV and to other nucleoside analogs. Since we used HSV-TK reporters with 3′ untranslated regions from either aldolase (TK reporter 1) or variant surface glycoprotein (VSG; TK reporter 2) genes (Fig. 1A), our screens also had the potential to identify loss of positive control operating via these distinct 3′ untranslated regions.

We established reporter strains expressing a BLA : HSV-TK fusion protein with 3′ untranslated regions from *aldolase* or *VSG*. BLA : HSV-TK expression was confirmed by protein blotting (Fig. S1A), while dose response assays revealed GCV half maximal EC_50_ values of 0.05 and 0.11 µg/mL, respectively (Fig. S1B and C). Both strains were used to assemble *T. brucei* RNAi libraries (Fig. 1A), as described (22). RNAi-based knockdown was induced in each library using tetracycline for 24 h prior to GCV-selection (Fig. 1A) and at two times the respective EC_50_ values. We observed an initial delay in growth under GCV selection, as expected, followed by an increased growth rate, in both cases (Fig. 1B). After 4 days of GCV selection, cultures were split and subjected to continued GCV selection at two or four times the EC_50_. We observed little difference between cultures selected at two or four times the EC_50_ over a further 4 days (Fig. 1B). Parasites were harvested for genomic DNA extraction from all four GCV-selected cultures at this point. We PCR amplified RNAi target fragments and deep sequenced the amplicons. Sequence reads were mapped to the *T. brucei* reference genome, and reads were quantified for each gene (Fig. 1C).

GCV must be phosphorylated in a three-step process to generate the active ganciclovir triphosphate (GCV-TP). HSV-TK is responsible for producing the monophosphate, guanylate kinase is responsible for producing the diphosphate, and nucleoside diphosphate kinase is responsible for producing the triphosphate. An analysis of the top hits (Fig. 1C; Data file S1) revealed guanylate kinase (Tb927.10.11700) and nucleoside diphosphate kinase [Tb927.11.16130 (23)], consistent with GCV-activation through multiple phosphorylation steps (Fig. 1D). Notably, genes that failed to register as hits in our screens included hits from prior RNAi screens using adenosine analogs for selection (10, 24): adenosine kinase (Tb927.6.2300), 4E-interacting protein (Tb927.9.11050), and a nucleoside transporter (Tb927.5.286b), all consistent with distinct activation and potentially also transport mechanisms for adenosine and guanosine analogs. Another nucleoside transporter (Tb927.2.6200) and a polyamine transporter (Tb927.9.10340), however, did feature as hits in our screens (Fig. 1C); these transporters may impact intracellular dNTP pools or GCV uptake. Taken together, these hits provided excellent validation for our screening approach.

### The top ganciclovir-sensitizing hits are nuclear HD82 and 6.2800

**Fig 2:**
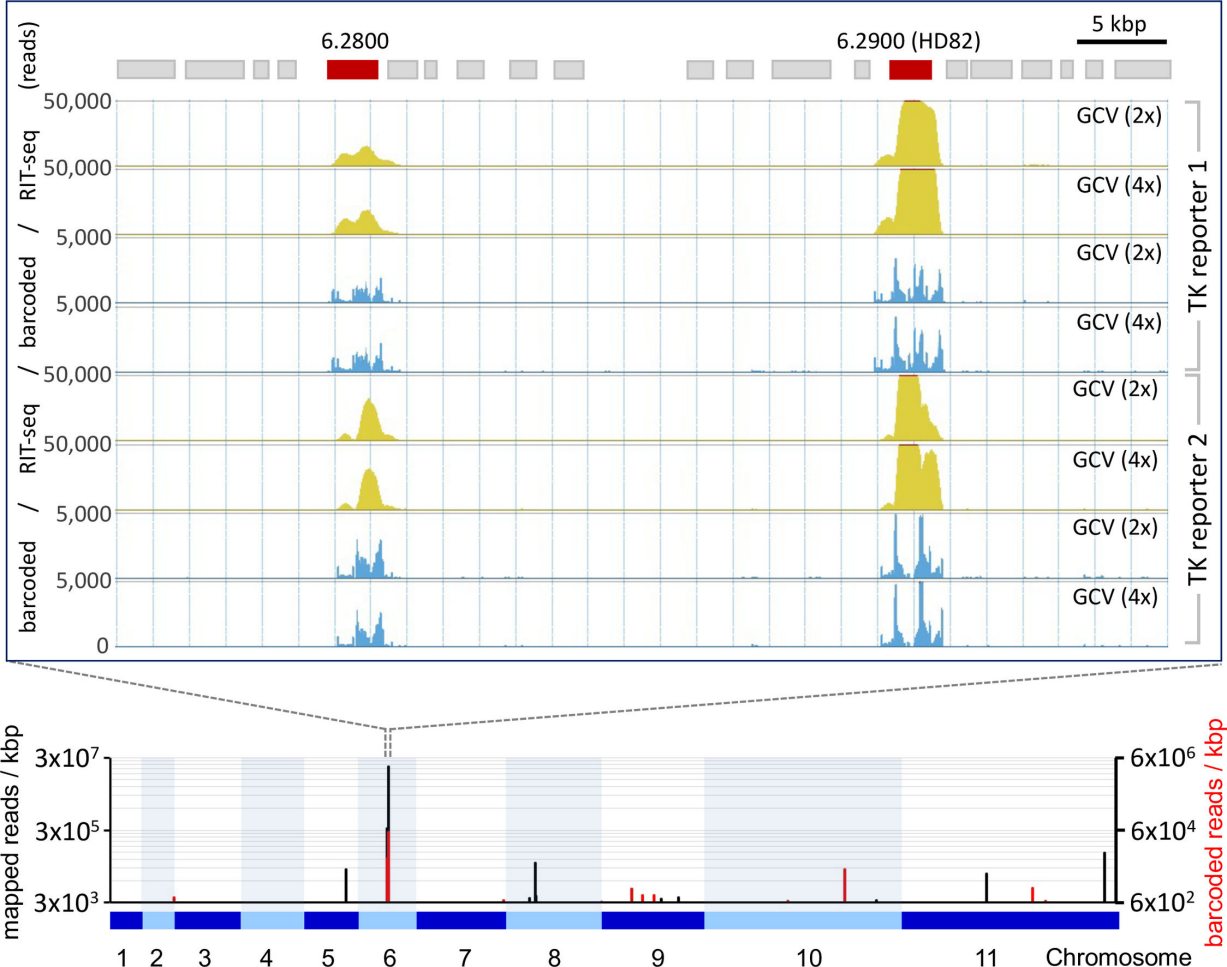
Two prominent GCV-sensitizing hits emerge from the RIT-seq screens. RIT-seq read mapping is shown for a 60-kbp region incorporating both the top two hits on chromosome 6. Total reads (yellow) and barcoded reads (blue) are shown; data from both reporter 1 and reporter 2 screens are shown. The lower panel is a genome-wide chromosomal map showing the barcoded reads over the 11 *T. brucei* megabase chromosomes, incorporating >7,400 genes. Total reads (black) and barcoded reads (red); data from the screen with reporter 1 and selection at 2× GCV EC_50_.

The two most dominant hits to emerge from our screens were “6.2800” and “6.2900” (Fig. 1C; Fig. 2). From the four experiments analyzed, we mapped an average of 2.5 ± 0.6 million RIT-seq reads to genes, with 6% ± 2% and 23% ± 5% of these mapped to 6.2800 and 6.2900, respectively. These two genes are within 28.3 kbp of each other, in the same polycistronic transcription unit on chromosome 6 (Fig. 2), although this does not necessarily reflect clustering of genes with related function; this polycistron incorporates approx. 120 putative protein-coding genes, with genes between the hits encoding an ATPase, a protein kinase, and a kinesin. Figure 2 depicts the relevant portion of the polycistronic unit and shows RIT-seq read mapping profiles from all four GCV-selected cultures. Since “barcoded” reads indicate the termini of RIT-seq fragments, we concluded that several RIT-seq fragments, from independent, multi-genome coverage *T. brucei* RNAi libraries, specifically and reproducibly identify these top two hits.

**Fig 3:**
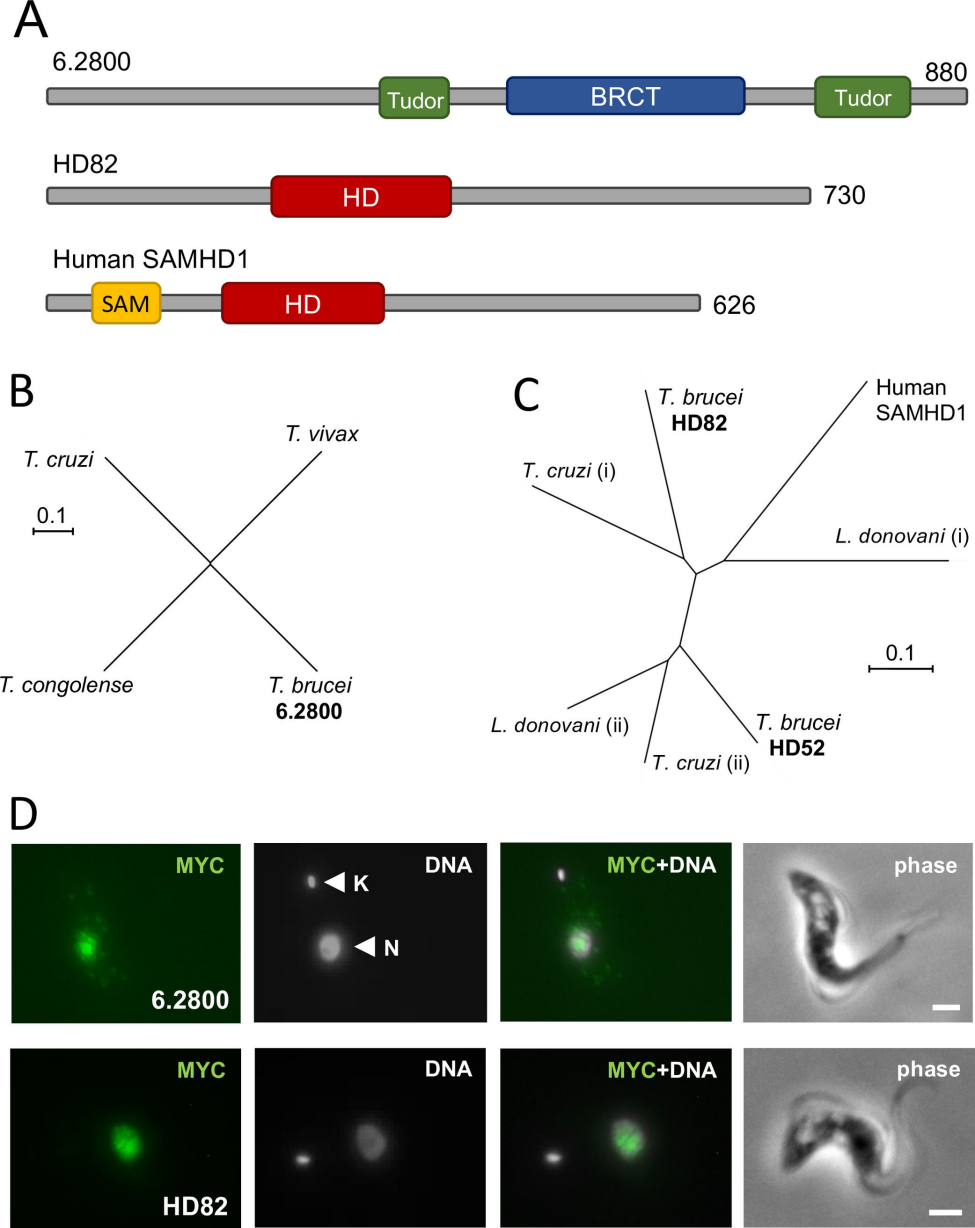
The top two hits HD82 and 6.2800 are nuclear proteins. (**A**) The schematic maps show protein domains identified in 6.2800, HD82, and human SAMHD1. (**B**) The unrooted phylogenetic tree shows *T. brucei* 6.2800-related proteins in the trypanosomes. *T. congolense*, TcIL3000_0_08110; *T. vivax*, TvY486_0602280; *T. cruzi*, TcCLB.511501.10. (**C**) The unrooted phylogenetic tree shows human SAMHD1 (NP_056289.2) and *T. brucei* HD82 and HD52 (Tb927.7.4810)-related proteins in the trypanosomatids; *T. cruzi*, TcCLB.507021.70 (i) and TcCLB.508579.10 (ii); *Leishmania donovani*, LdBPK_322680.1 (i) and LdBPK_060160.1 (ii). (**D**) Immunofluorescence microscopy localizes 6.2800^12MYC^ or HD82^12MYC^ to the *T. brucei* nucleus. DNA is stained with DAPI. K, kinetoplast; N, nucleus; scale bars, 2 µm.

While 6.2800 is uncharacterized and annotated as a “conserved hypothetical protein,” 6.2900 has been designated as HD82, an HD domain protein with a predicted mass of 82 kDa (25, 26). HD domains, named after the conserved histidine (H) and/or aspartate (D) residues, which are thought to coordinate divalent cations, are typically found in predicted dNTP phosphohydrolases (27). Examples include the human immunodeficiency virus restriction factor, SAMHD1 (28, 29) and *T. brucei* HD52 (Tb927.7.4810), which is a putative mitochondrial phosphohydrolase that controls pyrimidine homeostasis (26). The domain structures of 6.2800 and HD82, relative to SAMHD1, are depicted in Fig. 3A. The predicted mass of 6.2800 is 97 kDa, with two putative Tudor domains and a BRCT (BRCA1 C Terminus) domain. Tudor domains are often found in histone methylation “readers” (30), while BRCT domains are predominantly found in proteins involved in the DNA damage response (31). No additional domains were identified in HD82, beyond the HD domain. Indeed, HD82 differs from SAMHD1 in that it lacks a predicted SAM (sterile alpha motif), a protein interaction module.

A phylogenetic analysis revealed 6.2800 orthologs in other African trypanosomes and in the American trypanosome, *Trypanosoma cruzi* (Fig. 3B), but only a substantially more divergent protein in the other trypanosomatid, *Leishmania* (*E* = 2^−6^). HD82, and HD52, on the other hand, have conserved orthologs in *T. cruzi* and in *Leishmania* (Fig. 3C). To determine subcellular protein localization in *T. brucei*, we fused c-MYC epitope tags to one allele of each of these genes: at the N-terminus for HD82 and at the C-terminus for 6.2800. Both proteins migrated in gels as expected based on their predicted molecular mass (see below), and both proteins presented a punctate nuclear localization by immunofluorescence microscopy (Fig. 3D). Notably, SAMHD1 is also a nuclear-localized protein (32). Neither protein appeared to display major differences in abundance or localization during the cell cycle (Fig. S2).

### HD82 and 6.2800 sensitize *T. brucei* to purine analogs

**Fig 4:**
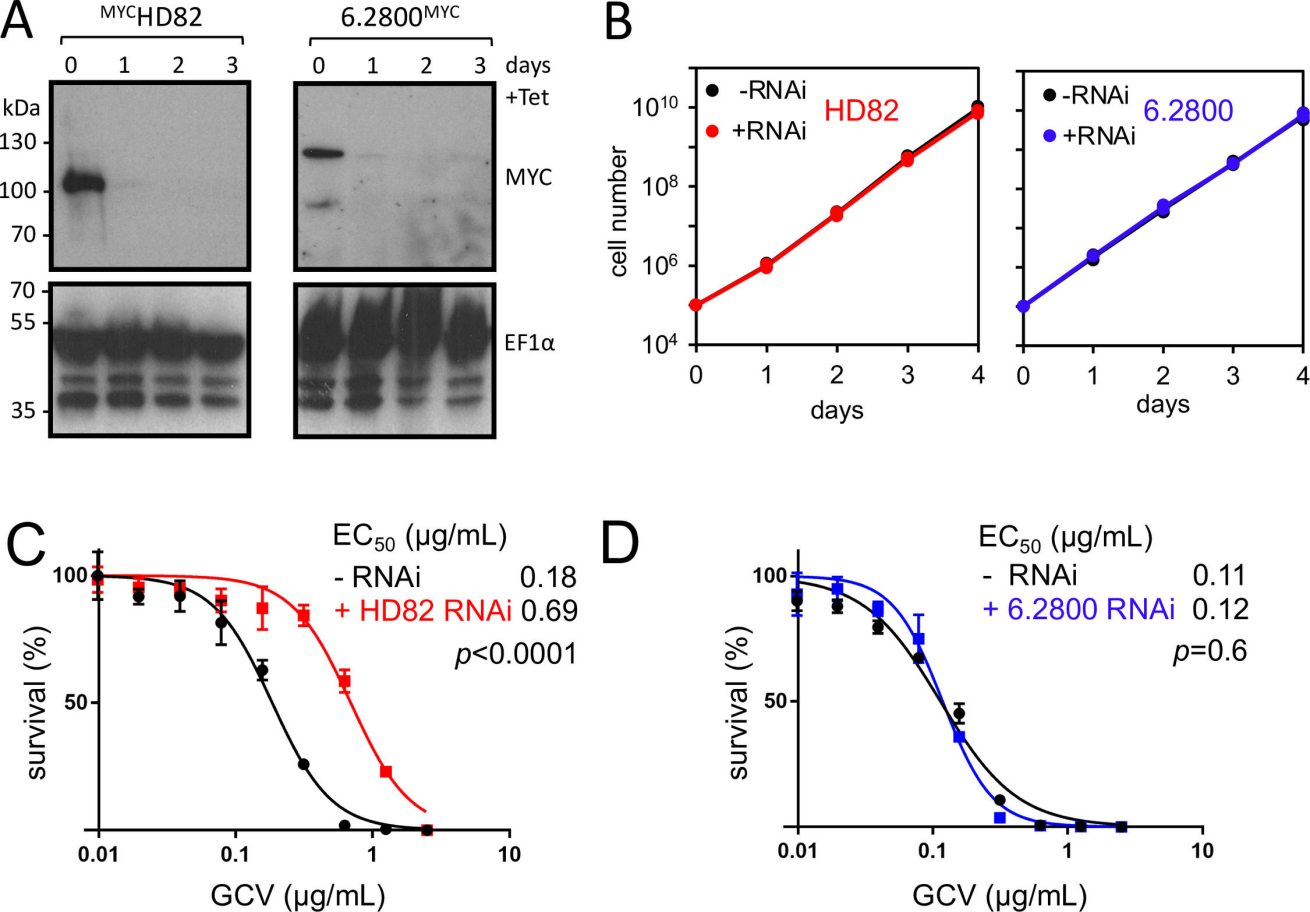
HD82 and 6.2800 knockdown and GCV dose response analysis. (**A**) The protein blots show effective and sustained knockdown of ^6MYC^HD82 or 6.2800^12MYC^. EF1α serves as a loading control. The estimated mass of ^6MYC^HD82 is 91 kDa and that of 6.2800^12MYC^ is 114 kDa. (**B**) The growth curves show exponential and unperturbed growth following either HD82 or 6.2800 knockdown. (**C**) GCV dose-response analysis before and after knockdown for HD82. (**D**) GCV dose-response analysis before and after knockdown for 6.2800. (**C, D**) Error bars, SD; three replicate assays. EC_50_ values and *P*-values (*F*-test) are shown.

To investigate GCV sensitization by HD82 and 6.2800 in more detail, we first generated inducible knockdown strains for each gene in a reporter 2 background. We also fused c-MYC epitope tags to one allele of each gene in these respective strains, to facilitate monitoring of knockdown. Efficient and sustained inducible knockdown was demonstrated by protein blotting in both cases (Fig. 4A). Neither knockdown was associated with a growth defect (Fig. 4B), consistent with prior observations (26, 33). Identification of both HD82 and 6.2800 as GCV-sensitizing hits in our RIT-seq screens, using HSV-TK-reporters with distinct 3′ untranslated regions, suggested that both proteins sensitized *T. brucei* to GCV in a manner that was independent of TK expression control by these 3′ untranslated regions. Consistent with this hypothesis, we observed no discernible change in expression level of the HSV-TK reporter following knockdown of either HD82 or 6.2800 (Fig. S3).

GCV selection was applied for 8 days in our RIT-seq screens, and we now sought to validate the HD82 and 6.2800 hits using a conventional dose response assay, conducted over 3 days of GCV selection. These assays confirmed that HD82 knockdown rendered *T. brucei* 3.8-fold resistant to GCV (Fig. 4C). The assays revealed no significant shift in GCV EC_50_ following 6.2800 knockdown, however (Fig. 4D). We, therefore, assessed GCV sensitivity after Tb927.6.2800 knockdown in a 6-day growth assay, which indicated that 6.2800 knockdown cells did indeed display increased tolerance to GCV selection at two times the EC_50_ (Fig. S4). These results are consistent with our RIT-seq profiles (Fig. 1C; Fig. 2) and with the idea that HD82 knockdown has the greatest impact on GCV resistance.

**Fig 5:**
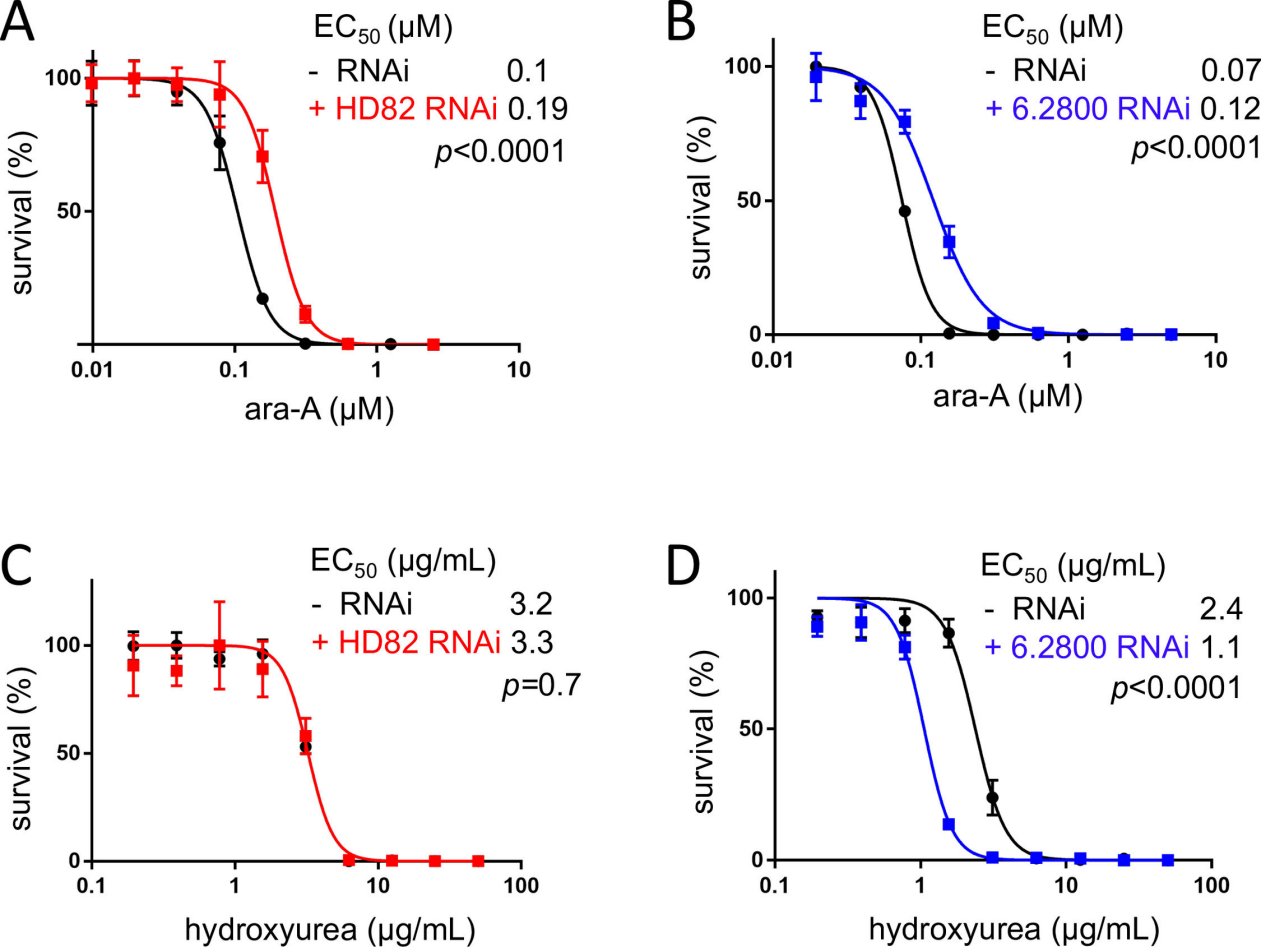
HD82 and 6.2800 sensitize *T. brucei* to purine analogs. (**A**) Dose response analysis for ara-A before and after HD82 knockdown. (**B**) Dose response analysis for ara-A before and after 6.2800 knockdown. (**C**) Dose response analysis for hydroxyurea before and after HD82 knockdown. (**D**) Dose response analysis for hydroxyurea before and after 6.2800 knockdown. Error bars, SD; three replicate assays. EC_50_ values and *P*-values (*F*-test) are shown.

Since GCV activity depends upon phosphorylation by ectopic expression of HSV-TK, we next tested an alternative purine analog that may be fully phosphorylated by native *T. brucei* kinase(s). In cells lacking HSV-TK, both HD82 and 6.2800 knockdown significantly increased the EC_50_ for adenine arabinofuranoside (ara-A or Vidarabine), an analog with the D-ribose replaced with D-arabinose (Fig. 5A and B). HD82 and 6.2800 may sensitize *T. brucei* to GCV and ara-A by limiting dNTP pools. To explore this hypothesis, we carried out dose-response assays with hydroxyurea, which itself reduces dNTP pools by inhibiting ribonucleotide reductase (34). Knockdown of HD82 revealed no significant shift in hydroxyurea EC_50_, while knockdown of 6.2800 did indeed render *T. brucei* hyper-sensitive to hydroxyurea (Fig. 5C and D).

### dATP appears to be the preferred substrate of the HD82 triphosphohydrolase

**Fig 6:**
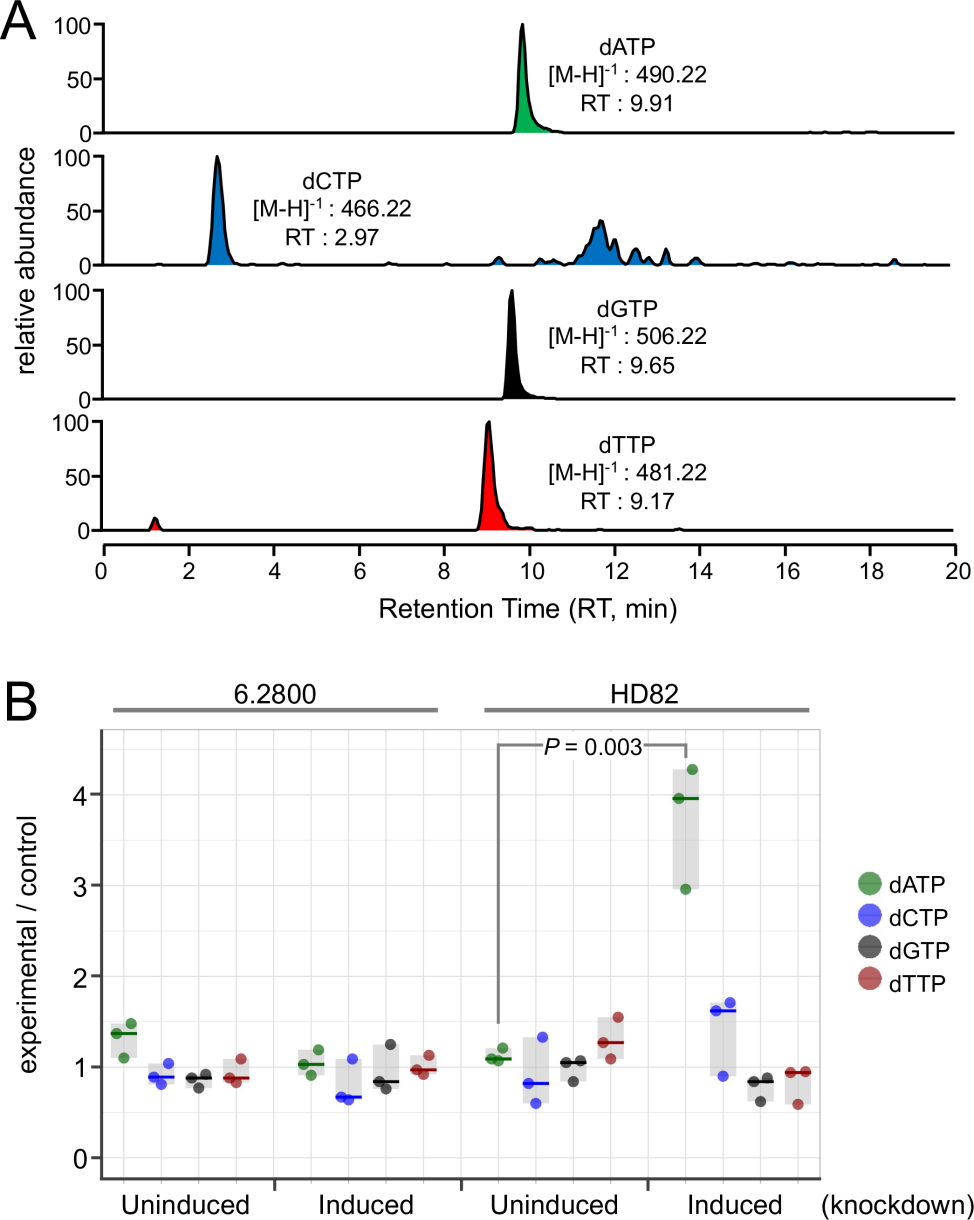
dATP abundance is specifically increased following HD82 knockdown. (**A**) The example data set shows the read-out of the LC-MS assay for dNTP abundance. (**B**) dNTP pools were quantified in *T. brucei* cell extracts before and after knockdown; *n* = 3 samples per condition. The horizontal lines indicate median values, and the gray boxes indicate the range of each data set. Heavy dATP was used as a reference in each sample, and values are expressed relative to a control parent *T. brucei* strain. Significance was assessed using an unpaired, two-tailed *t*-test.

To further probe the mechanisms by which 6.2800 and HD82 sensitize *T. brucei* to purine analogs, we established an assay to measure nucleotide pools before and after knockdown. We extracted nucleotides from these cells and assessed their abundance in triplicate for each sample using liquid chromatography and mass spectrometry (LC-MS). Heavy dATP was used as a reference in each sample, and the parent *T. brucei* strain was also analyzed and used as an unperturbed control. All four dNTPs were readily detected using this assay (Fig. 6A). Quantification of dNTPs in 6.2800 knockdown cells revealed no significant changes in dNTP pools (Fig. 6B). In contrast, quantification of dNTPs in HD82 knockdown cells revealed a significant and specific 3.3-fold increase in dATP abundance following knockdown (Fig. 6B). These results are consistent with the view that HD82 is indeed a triphosphohydrolase, with dATP as the preferred substrate; another recent study also found that dATP abundance was substantially increased following knockout of *T. brucei hd82* (25). Taken together with the results above, these findings suggest that HD82 sensitizes *T. brucei* to purine analogs by reducing purine pools, while 6.2800 sensitizes *T. brucei* to purine analogs by a distinct mechanism.

## DISCUSSION

We used genome-wide RNAi library screens to identify *T. brucei* genes that sensitize cells to nucleoside analogs. HD82 and Tb927.6.2800 emerged as the top two hits, both of which localized to the *T. brucei* nucleus. HD82 is a HD domain protein related to SAMHD1, a human viral restriction factor and triphosphohydrolase. Additional results suggest that HD82 and 6.2800 impact purine analog incorporation into DNA by distinct mechanisms. Our findings suggest that HD82 is a triphosphohydrolase that sensitizes cells to purine analogs by reducing the native dATP pool. In contrast, we suggest that the product of 6.2800, which incorporates a BRCT domain, sensitizes cells to purine analogs through DNA repair.

HD domains are typically found in predicted dNTP phosphohydrolases (27). Human SAMHD1 controls dNTP/deoxynucleoside homeostasis and thereby enhances nucleoside-analog efficacy against human immunodeficiency virus (35), presumably by depleting dNTPs and increasing the effective availability of nucleoside analog triphosphates. dNTP turnover is thought to dominate outside of S-phase during the cell cycle, when dNTP use is low (29, 36). A second *T. brucei* HD domain protein was previously found to localize to the mitochondrion and to control dTTP/dCTP pools; specifically, HD52 knockdown increased dCTP and decreased dTTP (26). Notably, HD52 was not a hit in our screens, consistent with specific and competitive inhibition of dGTP by GCV-TP. Additionally, GCV-TP is not a substrate for SAMHD1, while dGTP is (37). HD82 knockdown specifically increases dATP pools, which may explain resistance to the adenosine analog, ara-A, through competition for incorporation into DNA, but this does not readily explain resistance to GCV. Perhaps, unlike SAMHD1, HD82 also metabolizes GCV-TP.

Genome-wide RNAi library screens have been instrumental in revealing novel biology in *T. brucei* (38). Combining such screens with HSV-TK expression and negative selection presents opportunities for assessing further loss-of-function phenotypes, to identify positive regulators of gene expression, for example. Our findings should facilitate the analysis of such knockdown screens by pointing to those predictable GCV resistance mechanisms identified here. Finally, a prior study on nucleoside metabolism suggested that *T. brucei* encodes an unidentified 5′-nucleotidase that converts deoxypyrimidine nucleotides to their corresponding nucleosides (18). Tb927.9.10830 emerged as a candidate in that study, a gene that failed to register as a hit in our screens. We speculate that HD82 could fulfil this role. In summary, our results indicate that HD82 reduces nuclear dATP pools and sensitizes *T. brucei* to purine analogs, while 6.2800 sensitizes *T. brucei* to purine analogs by a distinct mechanism. These findings provide insights into nucleoside/nucleotide metabolism and nucleoside analog resistance mechanisms in trypanosomatids.

## MATERIALS AND METHODS

### *T. brucei* manipulation

Bloodstream-form *T. brucei* strain Lister 427 wild-type cells, 2T1 cells (39), and derivatives, including 2T1:T7, were used in this study. Cells were grown in HMI-11 media in a humidified incubator at 37°C and 5% CO_2_. Cells expressing an HSV-TK reporter were grown in media lacking additional thymidine, made by supplementing Iscove’s modified Dulbecco’s medium (IMDM; ThermoFisher) with 10% fetal bovine serum (FBS; Sigma), 1 mM hypoxanthine (Sigma), 0.05 mM bathocuproine disulfonic acid (Sigma), 1 mM sodium pyruvate (Sigma), and 1.5 mM L-cysteine (Sigma). Cell density was determined by counting cells on a hemocytometer (Hawksley). Transfection was carried out typically using 3–5 × 10^7^ cells at log phase (approx. 1 × 10^6^ cells/mL) in cytomix buffer using a Nucleofector (Amaxa) set on program X-001. Cells were selected using 2.5 µg/mL G418 (InvivoGen), 2.5 µg/mL hygromycin (Melford), or 10 µg/mL blasticidin (InvivoGen) as appropriate. RIT-seq library transfections were carried out as described previously (22) in 2T1:T7 strains containing an HSV-TK reporter.

### RIT-seq analysis

RIT-seq libraries were induced with tetracycline for 24 h prior to the addition of GCV (Sigma). Selected populations were harvested, and genomic DNA extracted using the DNeasy Blood and Tissue Kit (Qiagen). RIT-seq fragments were amplified from genomic DNA by PCR using Lib3F and Lib3R primers and Phusion DNA Polymerase (NEB). PCR amplicons were sequenced at the Beijing Genomics Institute using the DNB-seq platform. The FASTQ files with forward and reverse paired end reads were aligned to the reference genome v46 of *T. brucei* clone TREU927, reference sorted and indexed, quality evaluated, deduplicated, extracted, and parsed, with genome coverage data extracted, visualized, quantified, and normalized as described (40). The bash script containing the analysis pipeline, a Conda environment specification file for its execution, the python script to extract barcoded reads, and the versions of the software packages used are available in GitHub (https://github.com/mtinti/HD82-loss-of-function) and deposited in Zenodo 10.5281/zenodo.8093907.

### Plasmids

The initial reporter cassette, comprising a fusion of a blasticidin deaminase gene, a TY1 epitope tag, and the HSV-TK gene, with a VSG 3′ untranslated region, all flanked by β and α tubulin homology targeting fragments, was synthesized and cloned in a pMA-T vector (GeneArt). An aldolase 5′ untranslated region and neomycin phosphotransferase cassette were then added upstream of the reporter cassette. The VSG untranslated region was then excised using NotI and BglII and replaced with a PCR-amplified aldolase 3′ untranslated region. DNA fragments for transfection were excised from the plasmid by digestion with PmeI. The plasmid sequence can be found in Data file S1. RNAi target fragments were amplified and cloned into pRPa^iSL^ (41). HD82 was tagged with 6xMYC epitopes at the N-terminus using pNAT^6MYCX^ or with 12xMYC epitopes at the C-terminus using pNAT^X12MYC^, and 6.2800 was also tagged with 12xMYC epitopes at the C-terminus (41); these plasmids were linearized with KpnI, NdeI, or HpaI, respectively, prior to transfection. Oligonucleotide sequences can be found in Data file S1.

### Protein blotting

Bloodstream-form *T. brucei* (5 × 10^7^) were harvested and washed once with phosphate buffered saline (PBS) prior to lysis in Laemmli buffer at 1 × 10^6^ cells/µL. Ten microliters of lysate was loaded onto 10% SDS-PAGE gels (Bio-Rad). After protein separation, proteins were transferred onto nitrocellulose membrane (Millipore) using the Trans-blot Turbo (Bio-Rad) 10-min program and Tris/glycine transfer buffer (Bio-Rad). Membranes were blocked in 5% milk powder (Marvel) in PBS, then incubated with primary α-MYC at 1:5,000 (clone 9B11, Cell Signaling), washed with PBS 0.001% Tween 20 (Sigma), and incubated with secondary α-mouse HRP at 1:10,000 (Bio-Rad), followed by further washing. Signals were detected by incubation with ECL Prime (Amersham) and exposure to X-ray film (Amersham) before developing. Blots were stripped with western blot restore stripping buffer (ThermoFisher) before re-blocking and probing with α-eF1α (clone CBP-KK1, Millipore) as detailed above.

### Immunofluorescence microscopy

Bloodstream-form *T. brucei* (1 × 10^6^) were fixed in 3% paraformaldehyde in PBS (Sigma) for 10 min at RT before washing twice with PBS and settling onto poly-L-lysine slides (VWR) for 1 h at RT. Cells were permeabilized with 0.5% Triton X-100 (Sigma) in PBS for 15 min and blocked with 50% FBS (Sigma) in PBS for 15 min, followed by incubation with primary α-MYC (clone 9B11, Cell Signalling) at 1:5,000 in 3% FBS in PBS for 1 h at RT. Finally, slides were incubated with α-mouse Alexa Fluor-488-coupled secondary antibody (ThermoFisher) at 1:2,000 for 1 h at RT before VECTASHIELD (Vector Laboratories) with DAPI (4′,6-diamidino-2-phenylindole) was applied and a coverslip was used to seal on top. Z-stacks were captured using a Zeiss Axioscope Microscope and merged using the ImageJ Maximum Intensity Projection algorithm.

### Dose response assays

Cells were seeded in triplicate at 1 × 10^3^/ mL in a twofold serial dilution of either GCV (Sigma), hydroxyurea (Sigma), or ara-A (Sigma). The ara-A plate also contained 1 µM EHNA (Sigma) to prevent deoxyadenosine deamination (42). After 72-h incubation at 37°C, 20 µL resazurin sodium salt (alamarBlue, Sigma) was added at 0.5 mM in PBS to each well and incubated for a further 6 h. The fluorescence signal was determined using an Infinite 200 pro plate reader (Tecan) using an excitation wavelength of 540 nm and an emission wavelength of 590 nm. Data were analyzed, to derive EC_50_ values and statistics using Prism (GraphPad). RNAi knockdown strains were induced with 1 µg/mL tetracycline 24 h prior to plating.

### dNTP quantification by LC-MS

dNTPs were extracted as described (43). Briefly, 8 × 10^7^ *T. brucei* cells were washed in PBS and then lysed in 200 µL 0.5 M perchloric acid. One hundred nanograms of ^13^C and ^15^N labelled dATP (CK Isotopes) was spiked into each sample and used as an internal standard. Samples were centrifuged for 3 min at 17,600 × *g* at 4°C, and 100 µL supernatant was neutralized by the addition of 25 µL ice-cold 2.3 M potassium bicarbonate. Samples were centrifuged, and the supernatant was stored at −80°C. dNTP (dATP, dCTP, dGTP, dTTP, and heavy dATP) standards were used to optimize the LC-MS-based detection and quantification. dNTPs were measured in MRM mode with optimized collision energies and radio frequencies determined by infusing pure compounds. The TSQ Quantiva mass spectrometer was interfaced with an ultimate 3000 Liquid Chromatography System (Thermo Scientific). A PGC column (HyperCarb 30 × 1 ID 3µ Thermo Scientific) that was maintained at a constant temperature of 42°C was used for the separation. Mobile phase buffer A consisted of 0.3% (vol/vol) formic acid adjusted to pH 9.17 with ammonia prior to a 1/10 dilution. Buffer B was 80% (vol/vol) acetonitrile. The column was initially equilibrated with 3% buffer B for 11 min at a constant flow rate of 0.045 mL/min. Aliquots of 4 µL of each sample were loaded onto the column, and compounds were eluted with a linear gradient of 3%–5% buffer B in 2 min, 5%–80% buffer B in 2 min, and 80%–100% buffer B in 4 min, and the column was then equilibrated in 3% buffer B for 11 min. Eluents were sprayed into the TSQ Quantiva using an Ion Max NG ion source with the ion transfer tube with the temperature set at 350°C and vaporizer temperature at 30°C. The mass spectrometer was operated in negative mode spray voltage of 3,500, Sheath gas 40, Aux gas 10, and Sweep gas 2. One blank was run between each sample.

### Protein domain searches

Amino acid sequences of Human SAMHD1 and HD82 were searched for protein domains using InterPro (https://www.ebi.ac.uk/interpro/) (44). This failed to identify domains in 6.2800 so the Protein Databank file was downloaded from AlphaFold (https://alphafold.ebi.ac.uk [45, 46]) and uploaded into the Vector Alignment Search Tool (VAST) (https://www.ncbi.nlm.nih.gov/Structure/VAST/vastsearch.html) to identify protein domains.

## Data Availability

RIT-seq data are available at NCBI BioProject (Project ID PRJNA986443).

## SUPPLEMENTAL MATERIAL

Data File S1 msphere.00363-23-s0001.xls RIT-seq data, oligonucleotides, and reporter plasmid sequence.

Supplemental Figures msphere.00363-23-s0002.pdf Figures S1-S4.

ASM does not own the copyrights to Supplemental Material that may be linked to, or accessed through, an article. The authors have granted ASM a non-exclusive, world-wide license to publish the Supplemental Material files. Please contact the corresponding author directly for reuse.

## References

[1] Horn D. 2022. A profile of research on the parasitic trypanosomatids and the diseases they cause. PLoS Negl Trop Dis 16:e0010040. doi:10.1371/journal.pntd.0010040 35025891 PMC8758061

[2] Barnadas-Carceller B, Martinez-Peinado N, Gómez LC, Ros-Lucas A, Gabaldón-Figueira JC, Diaz-Mochon JJ, Gascon J, Molina IJ, Pineda de Las Infantas Y Villatoro MJ, Alonso-Padilla J. 2022. Identification of compounds with activity against Trypanosoma cruzi within a collection of synthetic nucleoside analogs. Front Cell Infect Microbiol 12:1067461. doi:10.3389/fcimb.2022.1067461 36710960 PMC9880260

[3] Bouton J, Furquim d’Almeida A, Maes L, Caljon G, Van Calenbergh S, Hulpia F. 2021. Synthesis and evaluation of 3'-fluorinated 7-deazapurine nucleosides as antikinetoplastid agents. Eur J Med Chem 216:113290. doi:10.1016/j.ejmech.2021.113290 33667845

[4] Bouton J, Maes L, Karalic I, Caljon G, Van Calenbergh S. 2021. Synthesis and evaluation of a collection of purine-like C-nucleosides as antikinetoplastid agents. Eur J Med Chem 212:113101. doi:10.1016/j.ejmech.2020.113101 33385837

[5] Hofer A. 2023. Targeting the nucleotide metabolism of Trypanosoma brucei and other trypanosomatids. FEMS Microbiol Rev 47:fuad020. doi:10.1093/femsre/fuad020 37156497 PMC10208901

[6] Hulpia F, Bouton J, Campagnaro GD, Alfayez IA, Mabille D, Maes L, de Koning HP, Caljon G, Van Calenbergh S. 2020. C6-O-alkylated 7-deazainosine nucleoside analogues: discovery of potent and selective anti-sleeping sickness agents. Eur J Med Chem 188:112018. doi:10.1016/j.ejmech.2019.112018 31931339

[7] Hulpia F, Campagnaro GD, Alzahrani KJ, Alfayez IA, Ungogo MA, Mabille D, Maes L, de Koning HP, Caljon G, Van Calenbergh S. 2020. Structure-activity relationship exploration of 3’-deoxy-7-deazapurine nucleoside analogues as anti-Trypanosoma brucei agents. ACS Infect Dis 6:2045–2056. doi:10.1021/acsinfecdis.0c00105 32568511

[8] Lin C, Hulpia F, Karalic I, De Schepper L, Maes L, Caljon G, Van Calenbergh S. 2021. 6-Methyl-7-deazapurine nucleoside analogues as broad-spectrum antikinetoplastid agents. Int J Parasitol Drugs Drug Resist 17:57–66. doi:10.1016/j.ijpddr.2021.08.001 34375904 PMC8358123

[9] Zenchenko AA, Drenichev MS, Il’icheva IA, Mikhailov SN. 2021. Antiviral and antimicrobial nucleoside derivatives: structural features and mechanisms of action. Mol Biol 55:786–812. doi:10.1134/S0026893321040105 34955556 PMC8682041

[10] Hulpia F, Mabille D, Campagnaro GD, Schumann G, Maes L, Roditi I, Hofer A, de Koning HP, Caljon G, Van Calenbergh S. 2019. Combining tubercidin and cordycepin scaffolds results in highly active candidates to treat late-stage sleeping sickness. Nat Commun 10:5564. doi:10.1038/s41467-019-13522-6 31804484 PMC6895180

[11] Reguera RM, Morán M, Pérez-Pertejo Y, García-Estrada C, Balaña-Fouce R. 2016. Current status on prevention and treatment of canine leishmaniasis. Vet Parasitol 227:98–114. doi:10.1016/j.vetpar.2016.07.011 27523945

[12] Kuhlmann FM, Robinson JI, Bluemling GR, Ronet C, Fasel N, Beverley SM. 2017. Antiviral screening identifies adenosine analogs targeting the endogenous dsRNA leishmania RNA virus 1 (LRV1) pathogenicity factor. Proc Natl Acad Sci U S A 114:E811–E819. doi:10.1073/pnas.1619114114 28096399 PMC5293060

[13] Valdés J, Taylor MC, Cross MA, Ligtenberg MJ, Rudenko G, Borst P. 1996. The viral thymidine kinase gene as a tool for the study of mutagenesis in Trypanosoma brucei. Nucleic Acids Res 24:1809–1815. doi:10.1093/nar/24.10.1809 8657559 PMC145877

[14] Lye L, Wang CC. 1996. Thymidine kinase as a Selectable marker for studying the biogenesis of glycosomes in Trypanosoma brucei. Exp Parasitol 82:211–217. doi:10.1006/expr.1996.0026 8617348

[15] Cross M, Taylor MC, Borst P. 1998. Frequent loss of the active site during variant surface glycoprotein expression site switching in vitro in Trypanosoma brucei. Mol Cell Biol 18:198–205. doi:10.1128/MCB.18.1.198 9418867 PMC121476

[16] Kim H-S, Li Z, Boothroyd C, Cross GAM. 2013. Strategies to construct null and conditional null Trypanosoma brucei mutants using Cre-recombinase and loxP. Mol Biochem Parasitol 191:16–19. doi:10.1016/j.molbiopara.2013.08.001 23954366 PMC3830529

[17] Ashton WT, Karkas JD, Field AK, Tolman RL. 1982. Activation by thymidine kinase and potent antiherpetic activity of 2'-Nor-2'-deoxyguanosine (2'NDG). Biochem Biophys Res Commun 108:1716–1721. doi:10.1016/s0006-291x(82)80109-5 6295389

[18] Leija C, Rijo-Ferreira F, Kinch LN, Grishin NV, Nischan N, Kohler JJ, Hu Z, Phillips MA. 2016. Pyrimidine salvage enzymes are essential for de novo biosynthesis of deoxypyrimidine nucleotides in Trypanosoma brucei. PLoS Pathog 12:e1006010. doi:10.1371/journal.ppat.1006010 27820863 PMC5098729

[19] Valente M, Timm J, Castillo-Acosta VM, Ruiz-Pérez LM, Balzarini T, Nettleship JE, Bird LE, Rada H, Wilson KS, González-Pacanowska D. 2016. Cell cycle regulation and novel structural features of thymidine kinase, an essential enzyme in Trypanosoma brucei. Mol Microbiol 102:365–385. doi:10.1111/mmi.13467 27426054

[20] Chello PL, Jaffe JJ. 1972. Isolation, partial purification, and properties of thymidine kinase from Trypanosoma (trypanozoon) brucei rhodesiense. J Parasitol 58:298–305. 5022867

[21] Fyfe JA, Keller PM, Furman PA, Miller RL, Elion GB. 1978. Thymidine kinase from herpes simplex virus phosphorylates the new antiviral compound, 9-(2-hydroxyethoxymethyl)guanine. J Biol Chem 253:8721–8727. 214430

[22] Glover L, Alsford S, Baker N, Turner DJ, Sanchez-Flores A, Hutchinson S, Hertz-Fowler C, Berriman M, Horn D. 2015. Genome-scale RNAi screens for high-throughput phenotyping in bloodstream-form African trypanosomes. Nat Protoc 10:106–133. doi:10.1038/nprot.2015.005 25502887

[23] Hunger-Glaser I, Hemphill A, Shalaby T, Hänni M, Seebeck T. 2000. Nucleoside diphosphate kinase of Trypanosoma brucei. Gene 257:251–257. doi:10.1016/s0378-1119(00)00401-7 11080591

[24] Mabille D, Cardoso Santos C, Hendrickx R, Claes M, Takac P, Clayton C, Hendrickx S, Hulpia F, Maes L, Van Calenbergh S, Caljon G. 2021. 4E interacting protein as a potential novel drug target for nucleoside analogues in Trypanosoma brucei. Microorganisms 9:826. doi:10.3390/microorganisms9040826 33924674 PMC8069773

[25] Antequera-Parrilla P, Castillo-Acosta VM, Bosch-Navarrete C, Ruiz-Pérez LM, González-Pacanowska D. 2023. A nuclear orthologue of the dNTP triphosphohydrolase SAMHD1 controls dNTP homeostasis and genomic stability in Trypanosoma brucei Front Cell Infect Microbiol 13:1241305. doi:10.3389/fcimb.2023.1241305 37674581 PMC10478004

[26] Yagüe-Capilla M, Castillo-Acosta VM, Bosch-Navarrete C, Ruiz-Pérez LM, González-Pacanowska D. 2021. A mitochondrial orthologue of the dNTP triphosphohydrolase SAMHD1 is essential and controls pyrimidine homeostasis in Trypanosoma brucei ACS Infect Dis 7:318–332. doi:10.1021/acsinfecdis.0c00551 33417760

[27] Aravind L, Koonin EV. 1998. The HD domain defines a new superfamily of metal-dependent phosphohydrolases. Trends Biochem Sci 23:469–472. doi:10.1016/s0968-0004(98)01293-6 9868367

[28] Ballana E, Esté JA. 2015. SAMHD1: at the crossroads of cell proliferation, immune responses, and virus restriction. Trends Microbiol 23:680–692. doi:10.1016/j.tim.2015.08.002 26439297

[29] Laguette N, Sobhian B, Casartelli N, Ringeard M, Chable-Bessia C, Ségéral E, Yatim A, Emiliani S, Schwartz O, Benkirane M. 2011. SAMHD1 is the dendritic- and myeloid-cell-specific HIV-1 restriction factor counteracted by Vpx. Nature 474:654–657. doi:10.1038/nature10117 21613998 PMC3595993

[30] Lu R, Wang GG. 2013. Tudor: a versatile family of histone methylation 'readers Trends Biochem Sci 38:546–555. doi:10.1016/j.tibs.2013.08.002 24035451 PMC3830939

[31] Gerloff DL, Woods NT, Farago AA, Monteiro ANA. 2012. BRCT domains: a little more than kin, and less than kind. FEBS Lett 586:2711–2716. doi:10.1016/j.febslet.2012.05.005 22584059 PMC3413754

[32] Rice GI, Bond J, Asipu A, Brunette RL, Manfield IW, Carr IM, Fuller JC, Jackson RM, Lamb T, Briggs TA, et al.. 2009. Mutations involved in aicardi-goutieres syndrome implicate SAMHD1 as regulator of the innate immune response. Nat Genet 41:829–832. doi:10.1038/ng.373 19525956 PMC4154505

[33] Alsford S, Turner DJ, Obado SO, Sanchez-Flores A, Glover L, Berriman M, Hertz-Fowler C, Horn D. 2011. High-throughput phenotyping using parallel sequencing of RNA interference targets in the African trypanosome. Genome Res 21:915–924. doi:10.1101/gr.115089.110 21363968 PMC3106324

[34] Chowdhury AR, Zhao Z, Englund PT. 2008. Effect of hydroxyurea on procyclic Trypanosoma brucei: an unconventional mechanism for achieving synchronous growth. Eukaryot Cell 7:425–428. doi:10.1128/EC.00369-07 18083826 PMC2238168

[35] Ordonez P, Kunzelmann S, Groom HCT, Yap MW, Weising S, Meier C, Bishop KN, Taylor IA, Stoye JP. 2017. SAMHD1 enhances nucleoside-analogue efficacy against HIV-1 in myeloid cells. Sci Rep 7:42824. doi:10.1038/srep42824 28220857 PMC5318888

[36] Chen Z, Hu J, Ying S, Xu A. 2021. Dual roles of SAMHD1 in tumor development and chemoresistance to anticancer drugs. Oncol Lett 21:451. doi:10.3892/ol.2021.12712 33907561 PMC8063254

[37] Arnold LH, Kunzelmann S, Webb MR, Taylor IA. 2015. A continuous enzyme-coupled assay for triphosphohydrolase activity of HIV-1 restriction factor SAMHD1. Antimicrob Agents Chemother 59:186–192. doi:10.1128/AAC.03903-14 25331707 PMC4291348

[38] Horn D. 2022. Genome-scale RNAi screens in African trypanosomes. Trends Parasitol 38:160–173. doi:10.1016/j.pt.2021.09.002 34580035

[39] Alsford S, Kawahara T, Glover L, Horn D. 2005. Tagging a T. brucei RRNA locus improves stable transfection efficiency and circumvents inducible expression position effects. Mol Biochem Parasitol 144:142–148. doi:10.1016/j.molbiopara.2005.08.009 16182389 PMC3833055

[40] Marques CA, Ridgway M, Tinti M, Cassidy A, Horn D. 2022. Genome-scale RNA interference profiling of Trypanosoma brucei cell cycle progression defects. Nat Commun 13:5326. doi:10.1038/s41467-022-33109-y 36088375 PMC9464253

[41] Alsford S, Horn D. 2008. Single-locus targeting constructs for reliable regulated RNAi and transgene expression in Trypanosoma brucei. Mol Biochem Parasitol 161:76–79. doi:10.1016/j.molbiopara.2008.05.006 18588918 PMC3828046

[42] Vodnala M, Fijolek A, Rofougaran R, Mosimann M, Mäser P, Hofer A. 2008. Adenosine kinase mediates high affinity adenosine salvage in Trypanosoma brucei. J Biol Chem 283:5380–5388. doi:10.1074/jbc.M705603200 18167353

[43] Ovens AJ, Gee YS, Ling NXY, Yu D, Hardee JP, Chung JD, Ngoei KRW, Waters NJ, Hoffman NJ, Scott JW, Loh K, Spengler K, Heller R, Parker MW, Lynch GS, Huang F, Galic S, Kemp BE, Baell JB, Oakhill JS, Langendorf CG. 2022. Structure-function analysis of the AMPK activator SC4 and identification of a potent pan AMPK activator. Biochem J 479:1181–1204. doi:10.1042/BCJ20220067 35552369 PMC9317966

[44] Paysan-Lafosse T, Blum M, Chuguransky S, Grego T, Pinto BL, Salazar GA, Bileschi ML, Bork P, Bridge A, Colwell L, et al.. 2023. Interpro in 2022. Nucleic Acids Res 51:D418–D427. doi:10.1093/nar/gkac993 36350672 PMC9825450

[45] Jumper J, Evans R, Pritzel A, Green T, Figurnov M, Ronneberger O, Tunyasuvunakool K, Bates R, Žídek A, Potapenko A, et al.. 2021. Highly accurate protein structure prediction with AlphaFold. Nature 596:583–589. doi:10.1038/s41586-021-03819-2 34265844 PMC8371605

[46] Varadi M, Anyango S, Deshpande M, Nair S, Natassia C, Yordanova G, Yuan D, Stroe O, Wood G, Laydon A, et al.. 2022. AlphaFold protein structure database: massively expanding the structural coverage of protein-sequence space with high-accuracy models. Nucleic Acids Res 50:D439–D444. doi:10.1093/nar/gkab1061 34791371 PMC8728224

