# Supplementary material for: Genome-wide screens connect HD82 loss-of-function to purine analog resistance in African trypanosomes
Source: mSphere. 2023 Dec 21;9(1):e00363-23. doi: 10.1128/msphere.00363-23 (PMC10826343; doi:10.1128/msphere.00363-23)
Supplement: Supplemental Figures — Figures S1-S4. [file msphere.00363-23-s0002.pdf]

# Genome-wide screens connect HD82 loss-of-function to purine analogue resistance in African trypanosomes

Anna Trenaman, Michele Tinti, Abdelmadjid Atrih and David Horn

Supplementary Figures 1-4

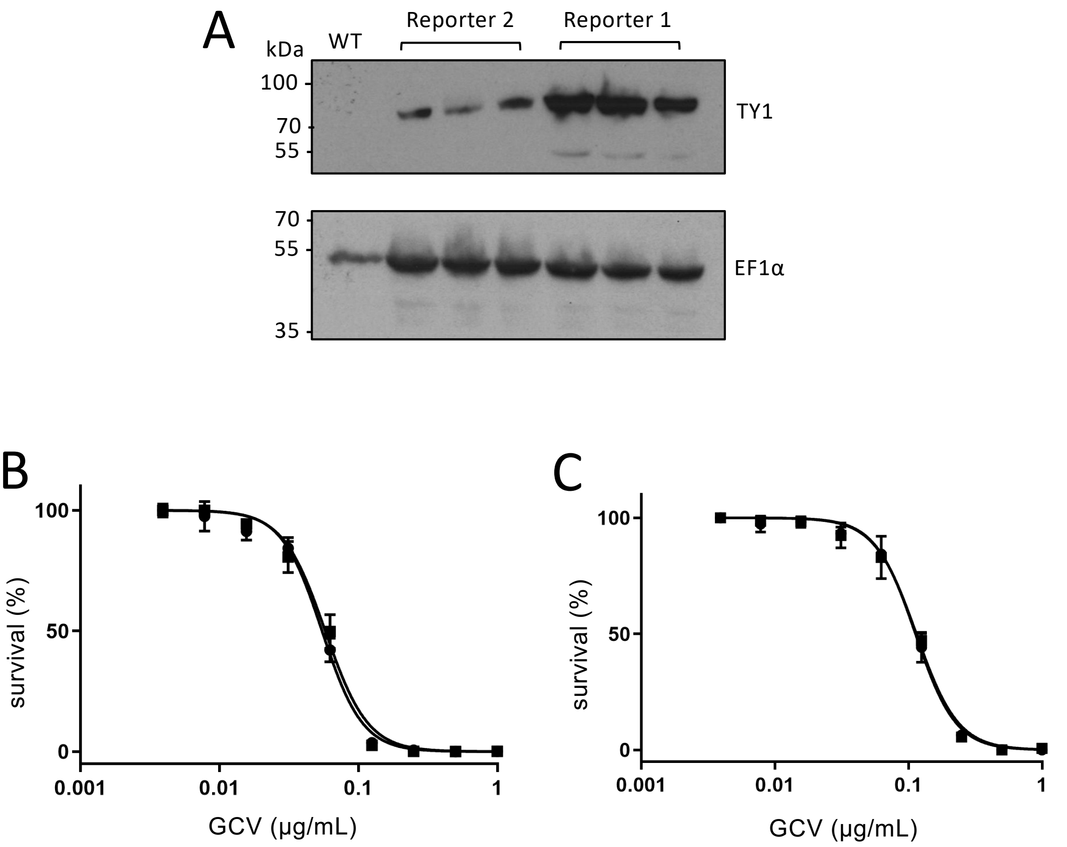


**Supplementary Figure 1. Assessment of BLA : HSV-TK reporter strains.**

(**A**) Protein blot showing BLA : HSV-TK reporter expression in reporter *T. brucei* strains compared to wild-type (WT) cells; the reporter has a centrally located TY1 tag. Reporter 1 has an *aldolase* 3’-untranslated region, while reporter 2 has a *VSG* 3’-untranslated region. EF1α served as a loading control. (**B-C**) GCV $EC_{50}$ values of 0.05 (*aldolase*) and 0.11 μg/mL (*VSG*) were derived using dose response assays. Error bars, SD; two biological replicates, each with three technical replicates.

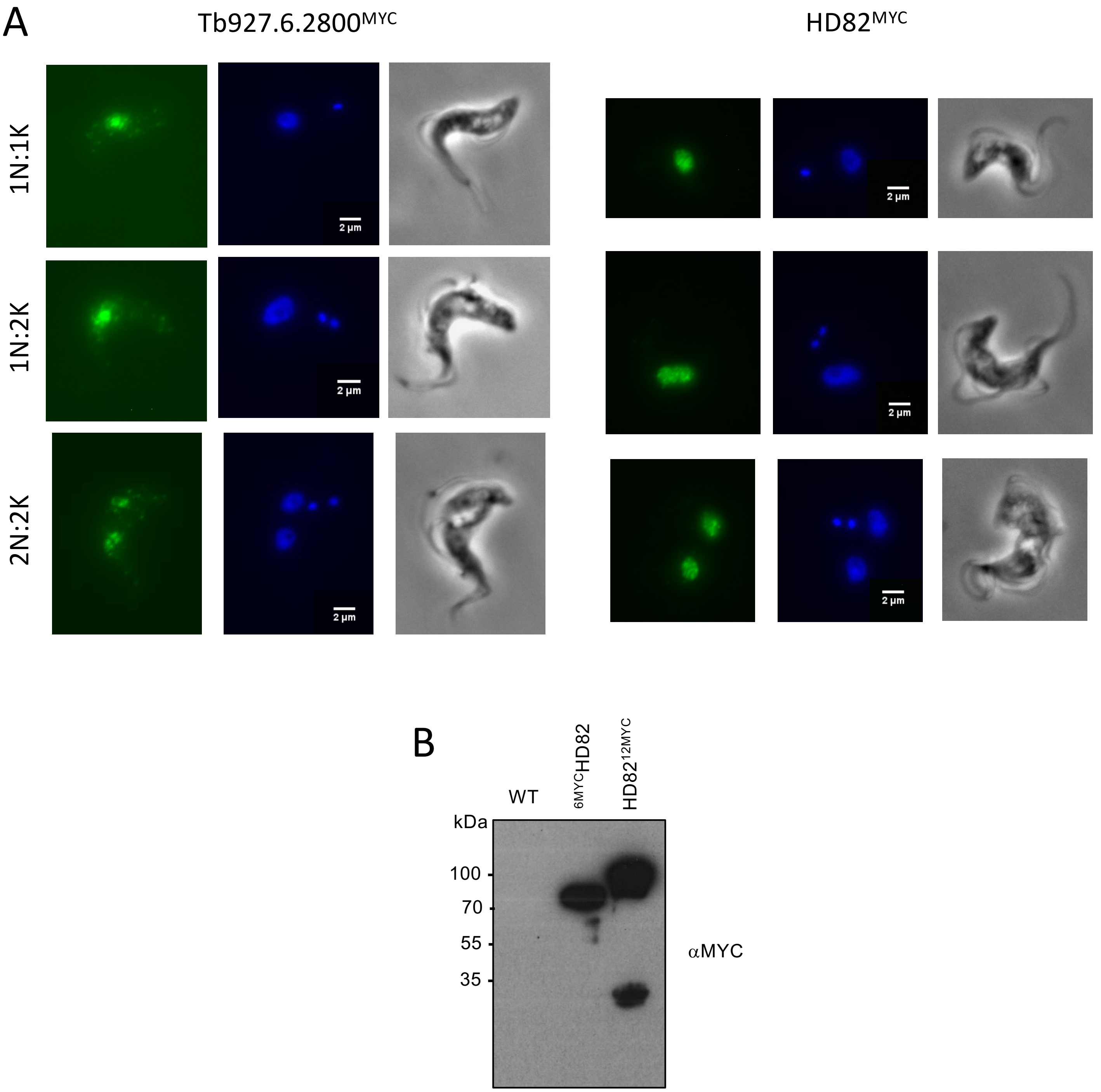


**Supplementary Figure 2. Assessment of subcellular localisation during the cell cycle.**

(**A**) Immunofluorescence microscopy localises $6.2800^{12MYC}$ or $HD82^{12MYC}$ to the *T. brucei* nucleus at different cell cycle stages; N, nucleus; K, kinetoplast; 1N:1K, G1; 1N:2K, G2M, 2N:2K, post-mitotic. DNA is stained with DAPI.; scale bars, 2 µm. (**B**) The protein blot shows migration of $^{6MYC}HD82$ or $HD82^{12MYC}$. WT, wild-type.

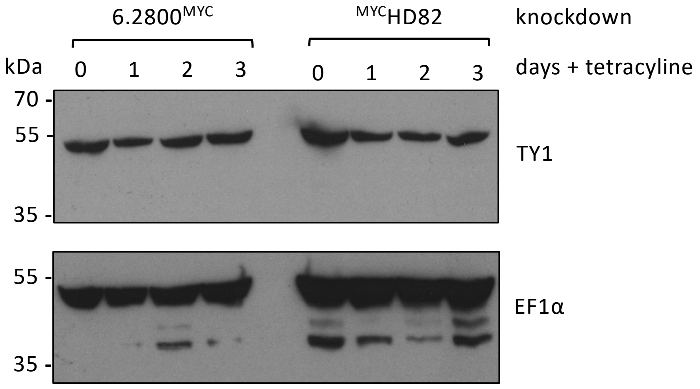


**Supplementary Figure 3. Reporter expression following knockdown of major screening hits.**

Protein blot showing BLA : HSV-TK reporter expression in reporter 2 *T. brucei* strains during 6.2800 or HD82 knockdown by RNAi (see Fig. 4A); the reporter has a centrally located TY1 tag. EF1α served as a loading control.

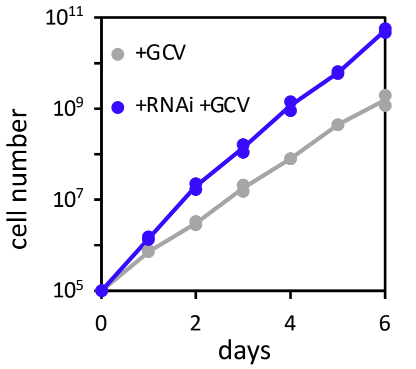


**Supplementary Figure 4. GCV-tolerance following 6.2800 knockdown.**

*T. brucei* growth in the presence of GCV (2 x $EC_{50}$), either with or without Tb927.6.2800 knockdown by RNAi.
